# Recurrence of preterm birth and perinatal mortality in northern Tanzania: registry-based cohort study

**DOI:** 10.1111/tmi.12111

**Published:** 2013-04-13

**Authors:** Michael J Mahande, Anne K Daltveit, Joseph Obure, Blandina T Mmbaga, Gileard Masenga, Rachel Manongi, Rolv T Lie

**Affiliations:** 1 Kilimanjaro Christian Medical University CollegeMoshi, Tanzania; 2 Department of Global Public Health and Primary Care, University of BergenBergen, Norway; 3 Centre for International Health, University of BergenBergen, Norway; 4 Norwegian Institute of Public HealthOslo, Norway; 5 Department of Obstetrics and Gynaecology, Kilimanjaro Christian Medical CentreMoshi, Tanzania

**Keywords:** preterm birth, recurrence risk, risk factors, perinatal death

## Abstract

**Objectives:**

To estimate the recurrence risk of preterm delivery and estimate the perinatal mortality in repeated preterm deliveries.

**Methods:**

Prospective study in Tanzania of 18 176 women who delivered a singleton between 2000 and 2008 at KCMC hospital. The women were followed up to 2010 for consecutive births. A total of 3359 women were identified with a total of 3867 subsequent deliveries in the follow-up period. Recurrence risk of preterm birth and perinatal mortality was estimated using log-binomial regression and adjusted for potential confounders.

**Results:**

For women with a previous preterm birth, the risk of preterm birth in a subsequent pregnancy was 17%. This recurrence risk was estimated to be 2.7-fold (95% CI: 2.1–3.4) of the risk of women with a previous term birth. The perinatal mortality of babies in a second preterm birth of the same woman was 15%. Babies born at term who had an older sibling that was born preterm had a perinatal mortality of 10%. Babies born at term who had an older sibling who was also born at term had a perinatal mortality of 1.7%.

**Conclusion:**

Previous delivery of a preterm infant is a strong predictor of future preterm births in Tanzania. Previous or repeated preterm births increase the risk of perinatal death substantially in the subsequent pregnancy.

## Introduction

Preterm birth is a major cause of morbidity and mortality among newborns and is estimated to account for 28% of neonatal mortality in the world each year (Lawn *et al*. 2010). World Health Organization (2012) estimates that around 15 million babies are born preterm each year, the majority in low-income countries. Hospital-based studies in sub-Saharan Africa have reported prevalences of preterm birth from 3.8% to 19.9% (Alhaj *et al*. 2010; Olusanya & Ofovwe 2010). Studies in Tanzania have reported prevalences between 10% and 16.7% (Fawzi *et al*. 2007; Habib *et al*. 2011). This is about twice as high as the figures reported in high-income countries (Lawn *et al*. 2010). Some risk factors for preterm birth in Africa and high-income countries are described (Beeckman *et al*. 2009; Jammeh *et al*. 2011). There is a strong association between preterm birth and perinatal mortality in Africa (Kinney *et al*. 2010).

Studies in high-income countries have reported high recurrence risk of preterm birth in subsequent pregnancies (Ananth *et al*. 2007; Mazaki-Tovi *et al*. 2007; McManemy *et al*. 2007; Esplin *et al*. 2008) and an association between recurrence of preterm birth and the risk of stillbirth and perinatal death (Melve *et al*. 1999; Surkan *et al*. 2004). The risk of perinatal death in a subsequent pregnancy has been associated with relative low birthweight of the previous infant (Skjaerven *et al*. 1988). There is, however, limited information on the recurrence risk of preterm birth and its implications on the risk of perinatal death in Africa.

There is a huge survival difference between preterm babies who are born in low-income countries compared to those who are born in high-income countries. This suggests that both the causes and the consequences of preterm birth may be different in low-income countries. Similarly, the causes and consequences of repeated preterm births in Tanzania may be different from the causes and consequences of repeated preterm births in high-income countries. In this prospective study, we estimate the recurrence risk of preterm delivery and estimate the perinatal mortality in repeated preterm deliveries in data from Tanzania.

## Methods

This study was conducted at Kilimanjaro Christian Medical Centre (KCMC), one of the four zone referral hospitals in Tanzania located in Moshi urban district, of the Kilimanjaro region in northern Tanzania. The centre primarily receives deliveries of women from the nearby communities, but also referred cases from other regions. On average, the hospital has approximately 3000 deliveries per year. The birth registry at KCMC has been in operation since July 2000. All delivering women are assigned a unique identity number at the hospital, and these numbers allowed the registry to link subsequent births of the same woman, even if these births occurred years apart.

The birth registry records information for all mothers who deliver in the obstetrics and gynaecology department. Trained midwives carried out daily interviews using a standardised questionnaire within 24 h of the delivery, or as soon as a mother has recovered. In addition, mothers admitted to the hospital were asked to provide their antenatal (ANC) and relevant data were abstracted. Data were also abstracted from the medical records in the hospital. A specially designed database system was used to register the data at the medical birth registry office.

Our primary outcomes were recurrence of preterm birth and perinatal death in subsequent pregnancies. Preterm birth was defined as a birth before 37 completed weeks of gestation. Gestational age was estimated based on menstrual period information and was recorded in completed weeks. We also estimated the risk of a preterm delivery after a previous delivery of a low birthweight baby (below 2500 g), previous delivery by induced labour or Caesarean section, or previous maternal infection or preeclampsia.

We used the unique maternal hospital identification numbers to link data on successive deliveries of the same woman. In order to reduce the likelihood of mismatch, the following validation procedure was used: (i) we matched the year of the first birth in the linked data with information on year of previous births recorded by interview questions for successive births; (ii) we calculated the birth interval from birth dates of the two births and checked against the recorded change in maternal age for the two deliveries. A discrepancy of more than 2 years led to exclusion from the linked data.

Our initial cohort consisted of 18 176 who gave birth to a singleton in their first recorded delivery at KCMC between 2000 and 2008. These were followed for consecutive births through 2010 in the registry. The follow-up was carried out by linking mothers' records for successive deliveries at KCMC using the unique maternal hospital identification numbers. The median follow-up period was 6.5 years. Women were excluded from the study if they had a delivery of multiples or had been referred from rural areas for various medical reasons in their first pregnancy.

**Figure 1:**
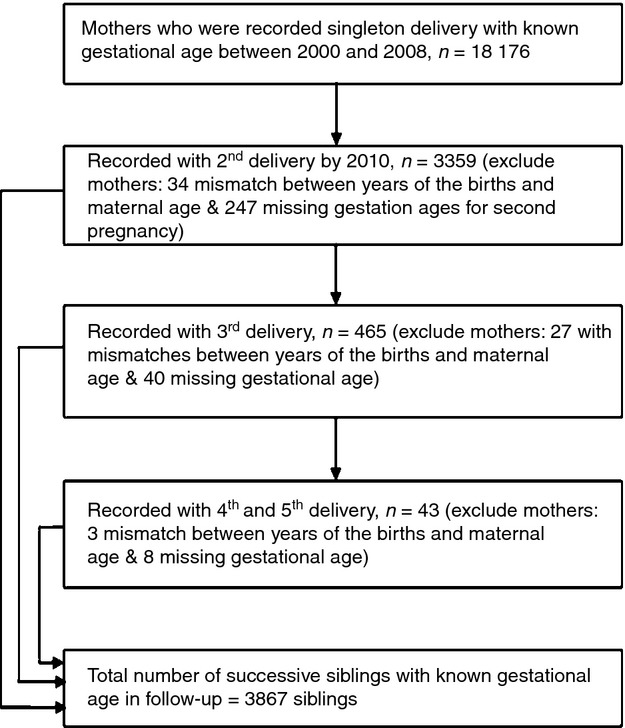
Schematic diagram for cohort estimation from Kilimanjaro Christian Medical Centre (KCMC) birth registry.

A total of 3359 (20%) women in the cohort were recorded with at least one more delivery within the follow-up period (Figure 1). We excluded 247 (7.4%) women from the analysis because their second birth lacked gestational age information. We estimated the recurrence of preterm birth from the first recorded birth of a mother to any of 3867 subsequent births.

We used reproductive history data obtained through interviews with the women to estimate that 32% of the women should have a second birth during study period. As we observed subsequent births for only 20%, the completeness of our follow-up of subsequent births was 63% (20/32). Data analysis was done using SPSS (SPSS Inc., version 18.0, Chicago, IL) and Stata 11.0. Means were compared using *t*-tests. Cross-tabulation was used to compare proportions between categorical variables using chi-square tests. We used mothers as the primary unit of analysis. A clustered analysis technique was employed, where births of the same mother formed a cluster, and robust variance estimates were used to take into account correlation between repeated births of the same mother. The relative risk of recurrence of preterm birth was estimated as measure of association between prior preterm delivery and preterm delivery in a subsequent pregnancy using a log-binomial regression model while adjusting for potentially confounding variables (maternal age and education attainment). A *P*-value <5% (2-tailed) was considered significant, and 95% confidence intervals (CI) of estimates were calculated.

The study was approved by the Kilimanjaro Christian Medical College (KCM-College) Research Ethics Committee. Informed verbal consent was obtained from participants before the interview and enrolment.

## Results

Among our final sample of 3359 women who had singleton deliveries and were recorded with subsequent births, 479 (14.3%) had preterm birth in their first recorded pregnancy. A lower percentage of 10.8% had preterm births among the women in our cohort who had no recorded subsequent births.

We compared baseline characteristics between women who had preterm birth and those with term births. Mothers' low education level, pre-eclampsia, a low birthweight infant, low BMI, poor attendance to ANC and a Caesarean section delivery were more likely to have a preterm delivery (Table 1).

**Table 1:** Socio-demographic characteristics of the 3359 women in the cohort at first birth who contributed more births in the study

Maternal characteristics (1st pregnancy)	Outcome in the 1st (or first recorded) pregnancy		

	Term birth (≥37 weeks)	Preterm birth (<37 weeks)	*P*-value*
Education level			0.004
≤12	1853	339 (15.5)	
12+	1019	140 (12.1)	
Body mass index†			<0.001
Underweight (<18.5 kg/m^2^)	389	92 (20.4)	
Normal (18.5–24.9 kg/m^2^)	262	28 (9.6)	
Overweight (25.0–29.9 kg/m^2^)	228	22 (8.8)	
Obese (≥30 kg/m^2^)	151	18 (10.7)	
Number of ANC visits			<0.001
<5	1596	363 (22.7)	
≥5	1284	116 (8.3)	
Maternal infections			0.64
Yes	1212	207 (14.6)	
No	1668	272 (14.0)	
Pre-eclampsia			<0.001
Yes	78	42 (35.0)	
No	2802	437 (13.5)	
Induced labour			0.01
Yes	1124	158 (12.3)	
No	1.756	321 (15.5)	
Caesarean section			<0.001
Yes	873	200 (18.6)	
No	2007	279 (13.4)	
Low birth weight			<0.001
Yes	338	243 (41.8)	
No	2542	236 (8.5)	
Perinatal death			<0.001
Yes	137	95 (40.9)	
No	2743	384 (12.3)	
Maternal age: mean (SD)	26.0 (4.9)	25.7 (5.3)	0.24
All women	2880	479 (14.3%)	

SD, Standard deviation.

* Chi-square tests for heterogeneity except of a *t*-test for mean maternal age.

† Numbers do not add to total because of missing values in the BMI variables.

Table 2 shows how conditions of the first recorded pregnancy were associated with the risk of preterm birth in a subsequent pregnancy. The absolute recurrence risk of preterm birth was 17.3%. Women with prior preterm birth had a 2.7-fold increased risk of preterm birth in their subsequent pregnancies compared with women who delivered an infant at term in the first pregnancy (95% CI: 2.1–3.4). Several other maternal and foetal conditions of the first pregnancy were also independently associated with increased risk of preterm birth in a next pregnancy. These include pre-eclampsia (RR = 2.5, 95% CI: 1.7–3.7), perinatal death (RR = 2.5, 95% CI: 1.9–3.5) and low birthweight (RR = 2.9, 95% CI: 2.3–3.6).

**Table 2:** Predictors for recurrence risk of preterm birth by maternal characteristics in the first pregnancy

Maternal characteristics**	*N*	Preterm birth in subsequent pregnancies			
		*n* (%)	RR (95% CI)*	Perinatal death *n* (%) *N* = 126	*P*-value
Gestational age					
Preterm birth	578	100 (17.3)	2.7 (2.1–3.4)	15 (15.0)	<0.001
Term birth	3289	202 (6.1)	Reference	11 (5.4)	
Pre-eclampsia					
Yes	152	26 (17.1)	2.5 (1.7–3.7)	5 (17.9)	<0.001
No	3715	276 (7.4)	Reference	21 (8.5)	
Perinatal death					
Yes	296	55 (18.6)	2.6 (1.9–3.5)	9 (16.4)	<0.001
No	3571	247 (6.9)	Reference	15 (6.1)	
Low birth weight					
Yes	592	106 (17.9)	2.9 (2.3–3.6)	21 (19.8)	<0.001
No	3275	197 (6.0)	Reference	6 (3.0)	
Caesarean section					
Yes	1238	111 (8.9)	1.2 (0.9–1.5)	8 (7.2)	0.13
No	2629	191 (7.3)	Reference	18 (9.4)	
Induced labour					
Yes	1514	101 (6.7)	0.8 (0.6–1.0)	9 (8.9)	0.1
No	2353	201 (8.5)	Reference	17 (8.5)	

RR, adjusted relative risk; CI, confidence interval.

* Adjusted for maternal age and maternal education in log-binomial model accounting for correlation between successive deliveries of the same mother.

** indicates maternal characteristics in the first pregnancy.

**Figure 2:**
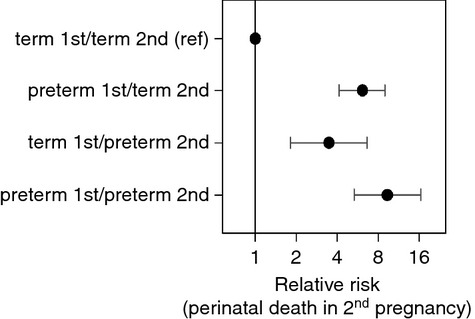
Recurrent preterm birth and subsequent risk of perinatal death.

Babies born to mothers who had a second preterm birth had a 9.2-fold (95% CI: 5.2–16.1) increased risk of perinatal death (Figure 2). Recurrence of preterm birth contributed 12% (15/126) of perinatal deaths among all subsequent births. A baby born at term, but who had an older sibling who was born preterm had a 5.9-fold (95% CI: 4.1–8.8) risk of perinatal death, while babies born preterm who had an older sibling born at term had a 3.4-fold risk (95% CI: 1.8–6.5). The reference category of babies born at term who also had an older sibling born at term had an absolute risk of perinatal death of 1.7%.

## Discussion

Our study shows that a history of preterm birth is a strong predictor of future preterm births and perinatal loss among Tanzanian women. The absolute recurrence risk of preterm birth estimated in our study of 17% was slightly lower than those estimated by Ananth *et al*. (2007) of 23% in Missouri and of Adams *et al*. (2000) of 26% among Black women in Georgia, USA.

Still, a previous preterm birth conferred a very high risk of perinatal mortality in the subsequent pregnancy in our data. Perinatal mortality was ninefold among babies born to mothers who had their second preterm birth. The risk remained high for babies born at term if the mother had a previous preterm birth (5.6-fold), while the risk was less than fourfold for babies born preterm if the mother had a previous term birth. This was in slight contrast with estimates of Melve *et al*. (1999) from Norway. They reported lower perinatal mortality in preterm births of mothers with a previous preterm birth than for mothers with a previous term birth. This difference could perhaps be explained by the differences in levels of perinatal mortality, causes of preterm birth, quality of antenatal care for high risk mothers and the quality of care of preterm babies between high- and low-income countries. We think repeated preterm birth in our data to a larger degree identifies a group of particularly vulnerable women and babies. In Western countries repeated preterm birth may signal that some women have a natural tendency of delivering preterm.

High child mortality after preterm births could hypothetically be attributed to shorter interpregnancy interval after a preterm birth. When we performed analyses with additional adjustment for interpregnancy interval, the estimates were virtually unchanged. Differences in interval are therefore not likely to contribute to the difference in risk.

Several other maternal and foetal conditions in the previous pregnancy such as pre-eclampsia, perinatal death and low birthweight were also associated with increased risks of preterm birth and high mortality in a subsequent pregnancy. This is consistent with other reports (Surkan 2004; Smith 2006; Gordon *et al*. 2012). The association between a previous delivery of a low birthweight baby and the risk of preterm birth and perinatal death in a subsequent pregnancy in our study may reflect the correlation between these factors and the value of using low birthweight as a proxy for preterm birth in Africa when information on gestational age is missing. The association between pre-eclampsia and risk of preterm birth and foetal death in subsequent pregnancies has also been reported by others (van Rijn *et al*. 2006; Lykke *et al*. 2009; Mahande *et al*. 2012). In our study, history of pre-eclampsia in a previous pregnancy increased the risk of preterm birth threefold. The high risk of preterm birth may be explained by recurrent pre-eclampsia, which increases the likelihood of early delivery due to other complication related to pre-eclampsia or the physicians' tendency for early interventions such as Caesarean section in subsequent pregnancies to prevent pre-eclampsia-related maternal risks. With the limited access to neonatal care in Tanzania, such practice may increase the risk for the baby.

Large cohort studies of recurrence risk of preterm birth are difficult to conduct in sub-Saharan Africa. An advantage of this study was the collection of extensive and standardised information on women's reproductive outcomes over more than a decade. Furthermore, relatively precise linkage of birth records enabled us to follow individual women through subsequent pregnancies, as long as they occurred at the same hospital. Our prospective design minimised problems of recall bias.

As our study was hospital based, it also had several limitations. First, our findings could be affected by selection bias by studying a group of women who could have different characteristics compared with women in the general population. Only 20% of the women who were defined into our cohort of 18 176 women returned for a subsequent pregnancy at the hospital within the median follow-up time of 6.5 years. We estimated that this represented 63% completeness in the follow-up of subsequent births. Many of the women had higher parity births and may have been at the end of their reproductive career. Still, our follow-up is likely to be incomplete, and women with medical problems may be over-represented in our data. Loss of follow-up may lead to biased estimates of the recurrence risk of preterm birth and perinatal death if women who came back to the hospital in their next births after a previous preterm birth had different risk characteristics from those whose deliveries were lost during the follow-up.

We excluded all women who were referred for medical reasons from our initial cohort. When we performed a subanalysis where we also excluded women who were referred for their subsequent births, the results were basically unchanged (a relative risk of 2.6 *vs*. 2.7 for recurrence of preterm birth). The consistency of our main results with other studies also suggests that our estimates are not very biased. Second, there could be errors in our record linkage. We did, however, exclude women who did not meet the additional matching criteria and those who missed records on gestational age, and the effect of this exclusion to our finding remains uncertain. Third, the estimation of gestational age was based on the recorded date of the last menstruation. Errors could lead to misclassification of preterm births. Finally, previous studies have found differences in recurrence risks after spontaneous and medically induced preterm births. Unfortunately, this difference and its possible effect on our results were not possible to assess in the present study.

## Conclusion

Our results confirm that preterm birth is a strong predictor for future preterm births also in Tanzania. Repeated preterm birth increases the risk of perinatal mortality and may cause reason for tighter clinical follow-up. Population-based studies would be important to verify the associations found in this study. An assessment of the value of targeted clinical follow-up was, however, not studied here and would require further investigation. Studies attempting to identify women who deliver preterm and may benefit from closer clinical follow-up may help in evaluating the clinical utility of the findings.
